# Salinity-Induced Anti-Angiogenesis Activities and Structural Changes of the Polysaccharides from Cultured *Cordyceps Militaris*

**DOI:** 10.1371/journal.pone.0103880

**Published:** 2014-09-09

**Authors:** Yangyang Zeng, Zhangrun Han, Peiju Qiu, Zijing Zhou, Yang Tang, Yue Zhao, Sha Zheng, Chenchen Xu, Xiuli Zhang, Pinghe Yin, Xiaolu Jiang, Hong Lu, Guangli Yu, Lijuan Zhang

**Affiliations:** 1 Key Laboratory of Marine Drugs, Ministry of Education, Shandong Provincial Key Laboratory of Glycoscience & Glycotechnology, School of Medicine and Pharmacy, Ocean University of China, Qingdao, China; 2 Department of Chemistry, College of Life Science and Technology, Jinan University, Guangzhou, China; 3 College of Food Science and Technology, Ocean University of China, Qingdao, China; Center for Cancer Research, National Cancer Institute, United States of America

## Abstract

Cordyceps is a rare and exotic mushroom that grows out of the head of a mummified caterpillar. Many companies are cultivating Cordyceps to meet the increased demand for its medicinal applications. However, the structures and functions of polysaccharides, one of the pharmaceutical active ingredients in Cordyceps, are difficult to reproduce *in vitro*. We hypothesized that mimicking the salty environment inside caterpillar bodies might make the cultured fungus synthesize polysaccharides with similar structures and functions to that of wild Cordyceps. By adding either sodium sulfate or sodium chloride into growth media, we observed the salinity-induced anti-angiogenesis activities of the polysaccharides purified from the cultured *C. Militaris*. To correlate the activities with the polysaccharide structures, we performed the ^13^C-NMR analysis and observed profound structural changes including different proportions of α and β glycosidic bonds and appearances of uronic acid signals in the polysaccharides purified from the culture after the salts were added. By coupling the techniques of stable ^34^S-sulfate isotope labeling, aniline- and D_5_-aniline tagging, and stable isotope facilitated uronic acid-reduction with LC-MS analysis, our data revealed for the first time the existence of covalently linked sulfate and the presence of polygalacuronic acids in the polysaccharides purified from the salt added *C. Militaris* culture. Our data showed that culturing *C. Militaris* with added salts changed the biosynthetic scheme and resulted in novel polysaccharide structures and functions. These findings might be insightful in terms of how to make *C. Militaris* cultures to reach or to exceed the potency of wild Cordyceps in future.

## Introduction

Cordyceps is a rare and exotic mushroom in that the fungus, either *Cordyceps Militaris* or *Cordyceps Sinensis*, grows out of the head of a mummified caterpillar habituating in Tibetan Plateau and the Himalayas between 3000 m and 5000 m above sea level [1]. Wild Cordyceps has been used for at least a thousand years in Traditional Chinese Medicine [2]. The annual market for Cordyceps-related products as herbal medicine or dietary supplements has been growing steadily not only in Asian but also in Western countries in last decade [3], [4], which makes wild Cordyceps worth far more than its weight in gold in world markets. Rarity, over-harvesting, and increased demand [5], [6], [7] had made wild Cordyceps an endangered species, which was declared by the Chinese authority of Convention on International Trade in Endangered Species (CITES) in 2012.

To spare wild Cordyceps and to bring its price within reach of average people, many companies are starting to cultivate Cordyceps. Most commercial products are mycelial powders of the fungi, *Cordyceps Militaris or Cordyceps Sinensis*, produced by liquid fermentation or fruiting bodies produced on artificial solid media. Even though growth conditions in artificial media are completely different from those *in insecta*, the cultured commercial products are usually considered to be similar or superior to those of wild Cordyceps in terms of the same or higher contents of established pharmacological active ingredients such as polysaccharides, cordycepin, ergosterols, and mannitol or cordyceptic acid [4].

Multiple biological activities, such as anti-inflammatory [8], anti-oxidant [9], anti-oxidative stress [10] anti-viral [11], immunomodulatory [12], hypoglycemic [13], anti-tumor [14] and anti-angiogenesis [15], have been reported for purified polysaccharides from Cordyceps, which make the polysaccharides one of the pharmaceutical active ingredients in Cordyceps. However, unlike the molecular structure-defined active ingredients of cordycepin, ergosterols and mannitol in Cordyceps, the chemical contentsof the polysaccharides in cultured commercial products quantified by colometric assays or monosaccharide composition analysis may not correlate with polysaccharide structures and biological functions.

Glycobiology is a field that is just entering the main stream of current molecular biology because of the discoveries of critical roles played by the genes responsible for N- and O-linked glycans [16] and polysaccharide [17] assembly during animal development and in human diseases. Polysaccharides are synthesized by all animals, plants, bacteria, fungi, and algae. Among them, the biosynthesis, biological functions, and roles in animal development of the polysaccharides have been extensively studied. It has been established that animal polysaccharides interact with a multitude of proteins including growth factors, growth factor receptors, chemokines, cytokines, proteases, protease inhibitors, etc. [17] and facilitate proper protein ligand/receptor interactions to initiate signal transduction events, and thereby regulate key processes in animal homoeostasis [18]. It has been recognized that the biosynthesis of animal polysaccharides is very different from that of DNA, RNA, and protein. There is no template but an array of polysaccharide assembling, modifying, and degrading enzymes along with monosaccharides, monosaccharide nucleotides, ATP (phosphate donor), PAPS (sulfate donor), polysaccharide binding proteins, and other organic/inorganic molecules coordinate polysaccharide biosynthesis, modification and degradation [19]. Interestingly, the expression of certain enzymes responsible for making special polysaccharide modifications may require the presence of specific inducers in order to express and to have functions [20]. As a result, animal polysaccharides have different monosaccharide compositions, linkage, modification, chain length, structure, and biological functions in a cell-, tissue-, species-specific manner. Moreover, epigenetic controls of multiple polysaccharide modification enzymes have been associated with different kinds of tumor growth [21], [22]. Therefore, animal polysaccharide biosynthesis is a process whereby a subset of genes dynamically responds to pivotal environmental changes to assemble specific polysaccharide structures to have specific functions.

The environmental control of polysaccharide biosynthesis and functions might be the case for fungi as well. It has been reported that fungi express very different transcriptomes when growing in different culture media [23], [24], which is especially true for *C. Militaris* [25]. The water-soluble constituents between wild and cultured Cordyceps are reported to be different as well [26]. These observations indicate that both transcriptomes and the biomolecules made by proteins translated from transcriptomes are affected by growth environment. Based on the knowledge gained from animal polysaccharide biosynthesis, we assumed that *C. Militaris* might make different polysaccharide with different biological functions when growing in different culture media.

When we observed that polysaccharides purified from the mushroom part of wild Cordyceps stimulated FGF/FGFR signals in a Baf3 cell-based model system [27], a property only associated with sulfated polysaccharides, we hypothesized that the salty environment of caterpillar bodies might contribute to the production of sulfated polysaccharides by the fungus. We assumed that mimicking the salty environment inside caterpillar bodies by adding salt to *C. Militaris* culture might be able to activate sulfotransferase genes as suggested by a previously published report. Unexpectedly, we discovered that sulfation was only one of the many changes in polysaccharides observed in our studies. Our data demonstrated that culturing *C. Militaris* with added salts changed the biosynthesis process and produced polysaccharides with novel structures and biological functions, which indicated that environmental factors play an important role in making functional polysaccharide structures. These findings were insightful in terms of how to evaluate the polysaccharide structures and functions and how to make cultured *C. Militaris* to achieve or to exceed the wild Cordyceps quality in future.

## Results

### Salinity-Induced Anti-Angiogenesis Activities of the Polysaccharides from Cultured C. Militaris

Fungi synthesize both water soluble and insoluble polysaccharides located in intracellular, cell wall, and extracelluar spaces serving energy storage, structure, and communication purposes. According to published reports, the water-soluble polysaccharides are the most active pharmacological components tested in over 300 kinds of polysaccharides extracted from either plants or fungi [28].

Wang *et al*. [29] extracted a water-soluble polysaccharide from cultured *C. militaris* containing galactose, mannose, glucose and glucuronic acid with the ratio of 1.00∶1.58∶7.89∶0.19. Yang *et al*. [30] obtained six polysaccharides from the cultured *C. militaris* by collecting different fractions in a gel filtration column. Subsequent studies indicated that one of the six polysaccharide, P70-1, had a backbone of (1→6)-linked β-D-mannose residues. The branches were mainly composed of (1→4)-linked α-D-glucose and (1→6)-linked β-D-galactose residues, and terminated with β-D-galactose and α-D-glucose residues. In contrast, Smiderle *et al*. [31] used a 5% KOH solution to extract and purify a polysaccharide. Subsequent analysis indicates this polymer is a glucogalactomannan, with a backbone of (1→2)-linked α-D-mannose, that can be substituted by (1→6)-linked-α-D-mannose or (1→2)-linked β-D-galactose side chains. It also has a three sugar side chain of α-D-mannose -(1→2)-linked-α-D-mannose-(1→6)-linked-β-D-glucose-(1→4). These published studies indicated that different purification methods can enrich different polysaccharide species not only with different monosaccharide compositions but also with distinct backbone/sugar linkages in *C. Militaris*. Thus it is still unclear how many types of polysaccharides the *C. Militaris* is capable of making. Since our goal is to test if salinity can induce novel *C. Militaris* polysaccharide structures and biological functions, we decided to purify and to study all water soluble polysaccharides from the *C. Militaris* cultures.

Previously established culture medium for *C. Militaris* has 6 g/L potato dextrose and 20 g/L glucose. One gram of potato dextrose usually contains ∼25 mg of salts, which means the culture medium has ∼150 mg/L salts. To avoid drastic perturbation of *C. Militaris* growth, we added 0, 0.05, 0.10, 0.25, 250, and 500 mg/L Na_2_SO_4,_ respectively; to the culture medium and let the *C. Militaris* grew for 10 days. We obtained almost the same amount of mycelium from each culture, which indicated that the added salts did not perturb the growth of the *C. Militaris*. We also obtained the similar amount of water soluble polysaccharides with an average yield of 20% from each de-lipided raw material (Table 1). We named the purified polysaccharides P0, P1, P2, P3, P4, and P5 accordingly, where P stands for Polysaccharides, 0 to 6 stands for 0, 0.05, 0.10, 0.25, 250, and 500 mg/L of Na_2_SO_4_ added cultures, respectively. The compatible C, H, N, and S compositions for each purified polysaccharide were observed by elemental analysis (Table 1). These data indicated that P0 to P5 were mainly carbohydrates as expected but P0 to P5 also contained small amounts of sulfur and nitrogen.

**Table 1:** Elemental analysis of P0-P5.

[Na_2_SO_4_] added to the *Cordyceps Militaris* culture	Yield(%)	Elemental Analysis(%)			
		C	H	N	S
0	22.7±1.2	37.2±1.4	5.74±0.19	1.51±0.05	0.52±0.01
0.05 mg L^−1^	23.7±0.9	37.7±1.2	5.44±0.12	1.67±0.04	0.46±0.02
0.10 mg L^−1^	17.6±1.0	37.64±1.5	6.69±0.31	1.55±0.06	1.15±0.03
0.25 mg L^−1^	17.3±0.6	38.18±1.3	6.77±0.21	1.39±0.07	1.03±0.01
250 mg L^−1^	18.9±0.7	38.6±1.1	6.87±0.19	1.51±0.05	0.78±0.01
500 mg L^−1^	22.3±1.0	39.26±1.2	6.86±0.18	1.74±0.04	1.19±0.02

**Figure 1:**
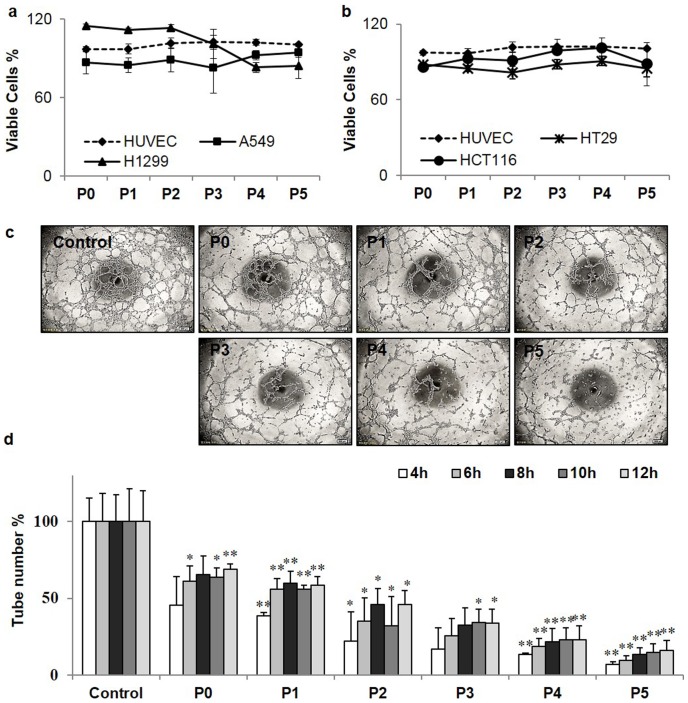
Growth inhibitory effect of P0-P5 (a and b) and the effects of P0-P5 on capillary tube formation of HUVECs on Metrigel (c and d). (a and b) Growth inhibitory effect of P0-P5 on A549, H1299, HCT116, HT29 and HUVEC cells. Two human lung cancer cell lines H1299 and A549 (a), two human colon cancer cell lines HT29 and HCT116 and HUVECs (Fig.1b) were used to measure the percentage of viable cells after 48 hrs exposure to 100 µg/ml P0-P5. The experiment was repeated three times with similar results. (c and d) Effects of P0-P5 on capillary tube formation of HUVECs on Metrigel. A 96-well plate coated with 55 µL Matrigel per well was allowed to solidify at 37°C for 30–60 min. HUVEC cells (3×10^4^ cells/well) were seeded and cultured in 200 µL F12 complete media containing 100 µg/mL of P0-P5 or blank control for 4–12 hrs. After 4, 6, 8, 10, 12 hrs of incubation, the enclosed capillary networks of tubes were photographed at 8 hrs (**c**) and the numbers of capillary tubes formed were counted (**d**). The experiment was repeated twice with comparable results. The untreated cells (control) were assigned values of 100 and the results were presented as mean ± S.D. (n = 4). Significance: *P<0.05, **P<0.01 vs. control group.

It was reported that the water soluble polysaccharides purified from *C. Militaris* inhibit microvascular formation [15]. We first checked if P0 to P5 had cytotoxicities towards cultured cells by adding 100 µg/ml P0 to P5 to two human colon cancer cell lines (HT29 and HCT116), two human lung cancer cell lines (H1299 and A549), and the human umbilical vein endothelial cells (HUVECs). The data in Fig. 1a&1b showed that the P0 to P5 at 100 µg/ml did not affect growth of any of the cells tested. We then examined if P0-P5 affected capillary tube formation of the HUVECs on the Matrigel. The pictures in Fig.1c were taken at 8 hrs after seeding the HUVECs on the Matrigel in the absence or presence of 100 µg/ml P0-P5. The data showed that the control HUVECs formed extensive capillary tubes whereas by adding100 µg/ml P0-P5 to the HUVECs on the Matrigel, the numbers of capillary tube formed were greatly reduced in the order of P5>P4>P3>P2>P1>P0> the control. The statistical data in Fig. 1d showed that the effects of P0-P5 peaked at 4 hrs, the numbers of the tubes remained about the same after 8 hrs, and the P0-P5 inhibited the capillary tube formation in a Na_2_SO_4_ concentration-dependent manner of the *C. Militaris* cultures used for the polysaccharide purification with statistical significance (*P<0.05, **P<0.01 vs. control group as shown in Fig. 1d).

All P0-P5 contained small amounts of sulfur according to the elemental analysis data shown in Table 1, which suggested that these polysaccharides might have sulfated residues. Since the sulfate in Na_2_SO_4_ could be used for sulfating polysaccharides in the *C. Militaris* culture, to distinguish salt- or sulfate-dependent anti-capillary tube formation activities of the polysaccharides, we grew the *C. Militaris* by adding increased amount of NaCl into the culture medium. The purified polysaccharides (P1C, P2C, P3C, P4C, and P5C) (Fig. S1 in File S1) from NaCl-added cultures also reduced the numbers of capillary tube formed in the order of P5C>P4C>P3C>P2C>P1C. Therefore, the anti-capillary tube formation activities of the polysaccharides were salinity- but not sulfate-dependent.

**Figure 2:**
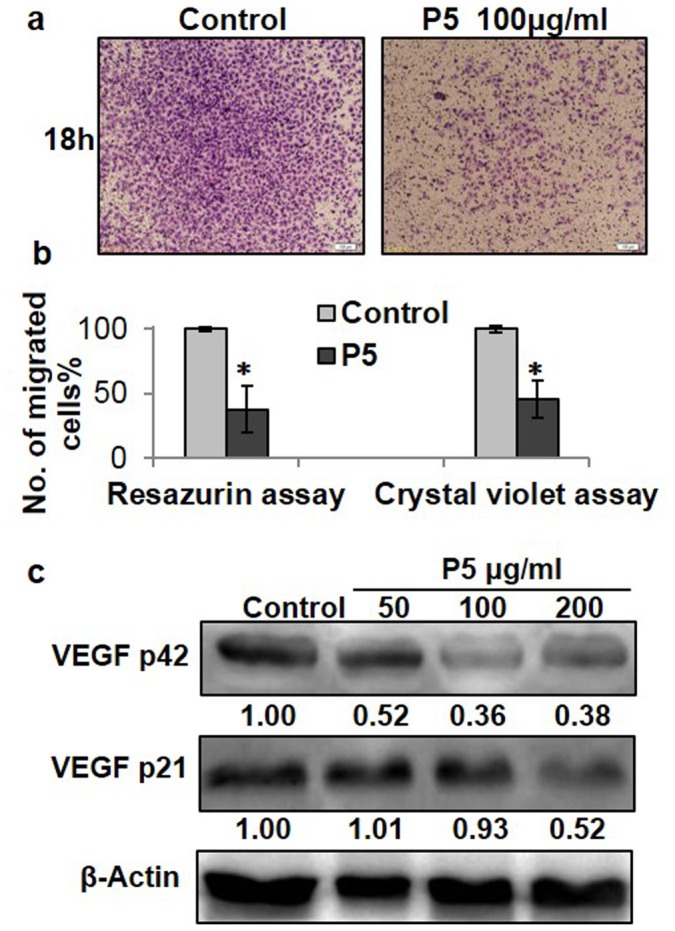
P5 reduced HUVEC cell migration and VEGF expression in HUVEC cells. P5 reduced HUVEC cell migration. The transwell assay was performed by using a 24-well chamber as the outer chambers and polycarbonate filters (8 µm pores) as the inner chambers as described in Methods. After incubation for 18 hrs at 37°C, the migrated cells on the lower surface of the filter were fixed in 90% ethanol and stained with 0.1% crystal violet. Images of the migrated cells were taken using a microscope (Olympus, CKX41, Japan) (**a**). The migrated cells were quantified at 595 nm after extraction with 10% acetic acid (**b**). Migrated cells in the outer chambers were also quantified independently by incubating the out chambers in F12 media with 10% FBS for 8 days followed by resazurin quantification (**b**). The untreated cells (control) were assigned values of 100 and the results were presented as mean ± S.D. (n = 3). Significance: *P<0.05 vs. control group. **Effects of P5 on the expression of VEGF in HUVECs.** The HUVEC cells were seeded in 6-well plate for 24 hrs, and then treated with P5 at 50 µg/ml, 100 µg/ml, and 200 µg/ml, respectively. After 48 hrs of incubation, cells were harvested and equal amount of cellular proteins were subject to Western immunoblotting as described in Methods. The numbers underneath of the blots represent band intensity (normalized to β-actin) measured by Image J software. β-Actin was served as an equal loading control (**c**). The experiments were repeated twice with similar results.

The capillary tube formation assay reflects both proliferation and migration abilities of endothelial cells. Since the purified polysaccharides of P0-P5 and P1C-P5C did not affect HUVEC growth (Fig. 1 and Fig. S1 in File S1), we further tested if P5 affected the HUVEC migration by employing the transwell cell migration assay. As shown in Fig. 2a, 100 µg/ml P5 greatly reduced the numbers of HUVECs migrated to the outer chamber of the transwell compared to that of the polysaccharide-free control. Crystal violet quantification indicated P5 inhibited ∼62% of HUVEC migration (Fig. 2b) whereas the resazurin quantification indicated P5 inhibited ∼55% cell migration (Fig. 2b). The two independent measurements confirmed that P5 inhibited the HUVEC cell migration significantly.

To understand the molecular mechanism underlying P5-induced effects on tube-formation and cell migration on HUVECs, we further tested if P5 had any effect on VEGF expression in HUVECs because VEGF plays a critical role in angiogenesis [32], [33]. The results in Fig. 2c showed that treating HUVECs with P5 down-regulated the VEGF expression in a P5 concentration-dependent manner as compared to the control. Therefore, the anti-angiogenesis effects of P5 observed both in the tube-formation (Fig. 1 & Fig S1 in File S1) and the cell migration assay (Fig. 2) might be resulted from down-regulated VEGF protein expression in the HUVEC-based system, which was consistent with literature that VEGF is the most powerful angiogenic factor in HUVECs.

### Salinity-Induced structural changes detected by ^13^C-NMR analysis

**Figure 3:**
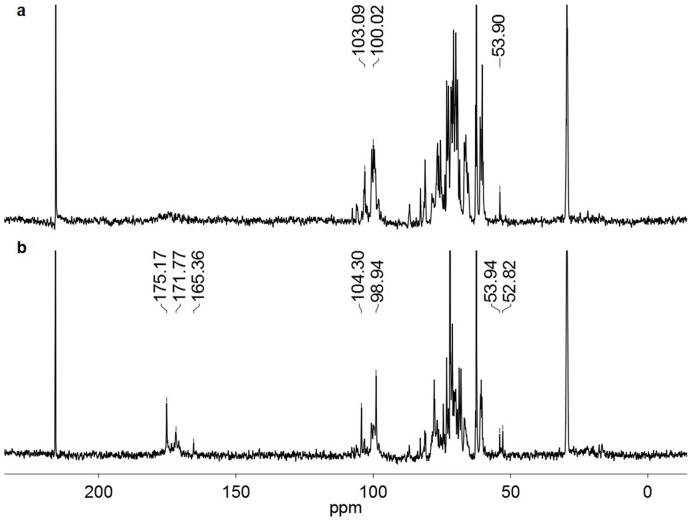
^13^C-NMR spectrum comparisons between P0 (a) and P5 (b).

According to published reports, the polysaccharides from *Cordyceps* mainly consist of mannose, glucose and galactose [11] with 1, 6-branched galactose in 1, 3-α-linked glucose and mannose backbone [34]. We performed the monosaccharide composition analysis of P0 to P6 (Fig. S2 in File S1) according to a published protocol [35]. Indeed, mannose, glucose and galactose were the three major monosaccharides present in P0 to P5. An increase in monosaccharides of glucosamine and glucose were observed in P5, but it was hard to correlate the minor changes in monosaccharide compositions with the increased anti-angiogenesis activities observed for the P0 to P5. Therefore we performed ^13^C-NMR analysis of P0 and P5. The spectra were shown in Fig. 3a (P0) and Fig. 3b (P5). ^13^C signals from 98 to 104 ppm represented the α or β linkages of the glycosidic bonds in polysaccharides. Apparently, P0 and P5 had very different proportions of α and β glycosidic bonds by direct comparison of the ^13^C signals in this region. Moreover, only P5 had ^13^C signals at 177.77, 175.19 and 165.36 ppm, which corresponded to the presence of –CO– of –COOH, –OCO– of –OCOCH_3_ and –NCO– of–NCOCH_3_ in the P5, which indicated the presence of hexosamine and uronic acid in the polysaccharides. Furthermore, the newly appeared ^13^C signal at 52.82 ppm in P5 ^13^C NMR spectrum also suggested the presence of –CH_3_ of –OCOCH_3_ or –CH_3_ of –NCOCH_3_ in the P5. In summary, salinity induced profound structural changes including different proportions of α and β glycosidic bonds and likely the presence of uronic acid and hexosamine residues in P5.

### The presence of polygalacturonic acid in P5 detected by mass spectrometry (MS) and capillary high performance liquid chromatography-coupled MS (LC-MS) analysis

**Figure 4:**
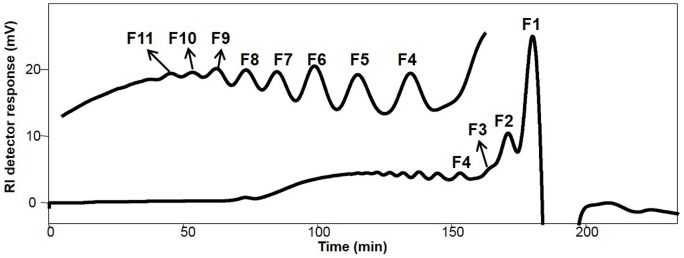
Preparation of oligosaccharides fractions 1-11 (F1-F11) from P5 by partial acid hydrolysis. The P5 hydrolysis was carried out by using 0.05 M TFA at 100°C for 1.5 hrs. The hydrolysates were subjected to gel filtration chromatography using a Superdex 30 HPLC column. A 0.1 M NH_4_HCO_3_ solution at a flow rate of 0.1 mL/min was used for oligosaccharide elution. Each oligosaccharide fraction (F1 to F11) was monitored by an online refractive index detector and collected accordingly.

**Figure 5:**
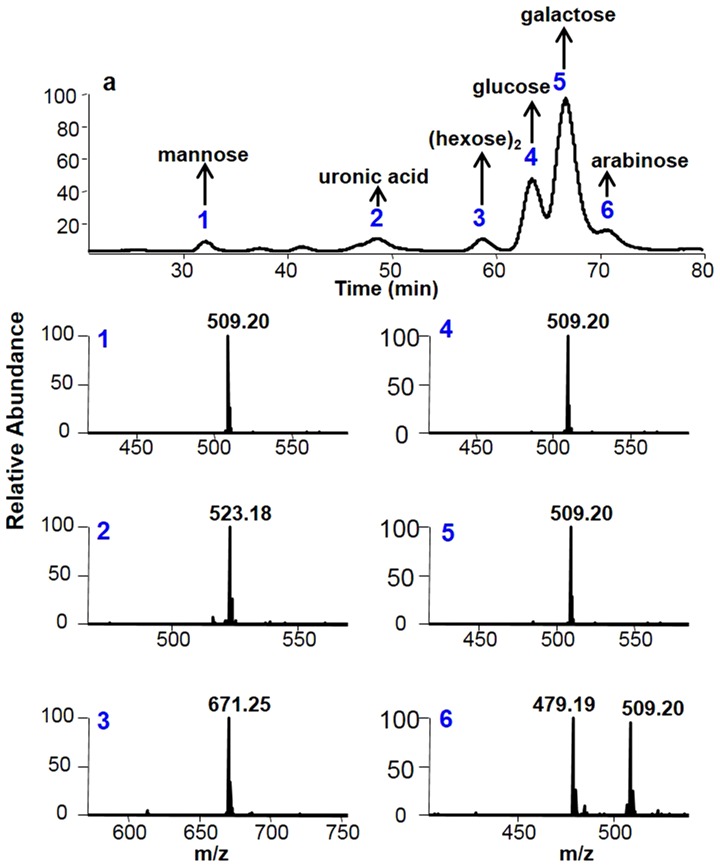
The mono- and disaccharide analysis of fraction 1(F1) by PMP labeling followed by capillary HPLC-MS analysis. The mono- and disacahrides in F1 was labeled with PMP and analyzed by capillary HPLC-MS (Details is in Methods).

The ^13^C NMR analysis indicated the presence of uronic acid in P5 but the monosaccharide composition analysis of P5 by using a published protocol did not detect the presence of uronic acid. We suspected that the harsh hydrolysis conditions used for the monosaccharide composition analysis might destroy the uronic acid and make it undetectable. Indeed, even in animal polysaccharides consisting of uronic acid and hexocosamine repeating disaccharides, uronic acid is barely detectable after acid hydrolysis. Thus, we decided to partially hydrolyze the P5. We used 0.05 M trifluoroacetic acid (TFA) at 100°C for 4 hrs to hydrolyze P5. The partially hydrolyzed products were then separated by a sizing column as shown in Fig. 4 where fractions 1 to 11(F1 to F11) were collected for further analysis. It turned out the F1 was composed of mainly monosaccharides after tagging it with 1-phenyl-3-methyl-5-pyrazolone (PMP) [35] followed by using our newly developed LC-MS method for monosaccharide composition analysis (Fig. 5). Five monosaccharides and one hexose disaccharide were detected by both LC and MS analysis (1: mannose, 2: uronic acid, 3: (hexose)_2_, 4: glucose, 5: galactose, and 6: arabinose) by comparing to the elution positions and m/z of PMP-derived monosaccharide standards. Comparing to the data shown in Fig. 5, galactose and glucose, but not mannose, were the most abundant monosaccharides after partial hydrolysis, indicating galactose and glucose might be located at the branching positions of the P5. The branching monosaccharides was easily hydrolyzed into monosaccharides whereas the mannose might formed the backbone of the P5, which made it harder to be hydrolyzed into monosaccharide.

By directly injecting F1 to F11 into MS followed by detecting the negatively charged ions, we obtained a large quantity of MS data from each fraction. Interestingly, by comparing calculated with theoretical molecular masses, it was quite obvious that F1 to F11 contained polyuronic acid with or without methyl (CH_3_) or acetyl (CH_3_CO) modifications ranged from 2 to 11 uronic acid in addition to a few sulfated neutral oligosaccharides as shown in Table 2.

**Table 2:** Observed polygalacturonic acids and sulfated oligosaccharides from fractions 1-11 (F1-F11).

Fractions	Ions found (charge)	Calculated molecular mass	Structure	Theoretical molecular mass
**F1**	193.04(−1)	194.04	L	194.04
	215.03(−1)	216.03	L+Na	216.03
	225.06(−1)	226.06	L+H_2_O+CH_3_	226.07
**F2**	184.03(−2), 369.07(−1)	370.06	2L	370.07
	355.09(−1)	356.09	H+L	356.09
**F3**	272.05(−2)	546.1	3L	546.11
	279.05(−2)	560.1	3L+CH_3_	560.12
	284.05(−2)	570.1	3L+COCH_3_-H_2_O	570.1
	291.1(−2)	584.2	3H+SO3	584.13
**F4**	239.71(−3), 360.06(−2)	722.13	4L	722.14
	244.38(−3), 367.07(−2)	736.14	4L+CH_3_	736.15
	272.05(−2)	546.1	3L	546.1
	372.07(−2)	746.14	4H+SO3	746.18
**F5**	239.71(−3), 360.06(−2)	722.13	4L	722.14
	263.04(−2)	528.08	3L-H_2_O	528.1
	272.05(−2)	546.1	3L	546.1
	298.38(−3), 448.08(−2)	898.14	5L	898.17
	455.09(−2)	912.18	5L+CH_3_	912.19
**F6**	213.83(−5), 267.54(-4), 357.06(−3), 536.09(−2)	1074.15	6L	1074.2
	298.38(−3), 448.08(−2)	898.14	5L	898.17
	361.73(−3)	1088.19	6L+CH_3_	1088.22
**F7**	249.04(−5), 311.55(−4), 415.74(−3), 624.11(−2)	1250.2	7L	1250.24
	267.54(−4), 357.06(−3), 536.09(−2)	1074.16	6L	1074.2
	420.41(−3)	1264.23	7L+CH_3_	1264.25
	423.06(−3)	1272.18	7L+Na	1272.22
	474.42(−3)	1426.26	8L	1426.26
	479.09(−3)	1440.27	8L+CH_3_	1440.28
	483.76(−3)	1454.28	8L+2CH_3_	1454.3
**F8**	236.7(−6), 284.25(−5), 355.56(−4), 474.42(−3)	1426.2	8L	1426.26
	249.04(−5), 311.55(−4), 415.74(−3)	1250.2	7L	1250.24

**Figure 6:**
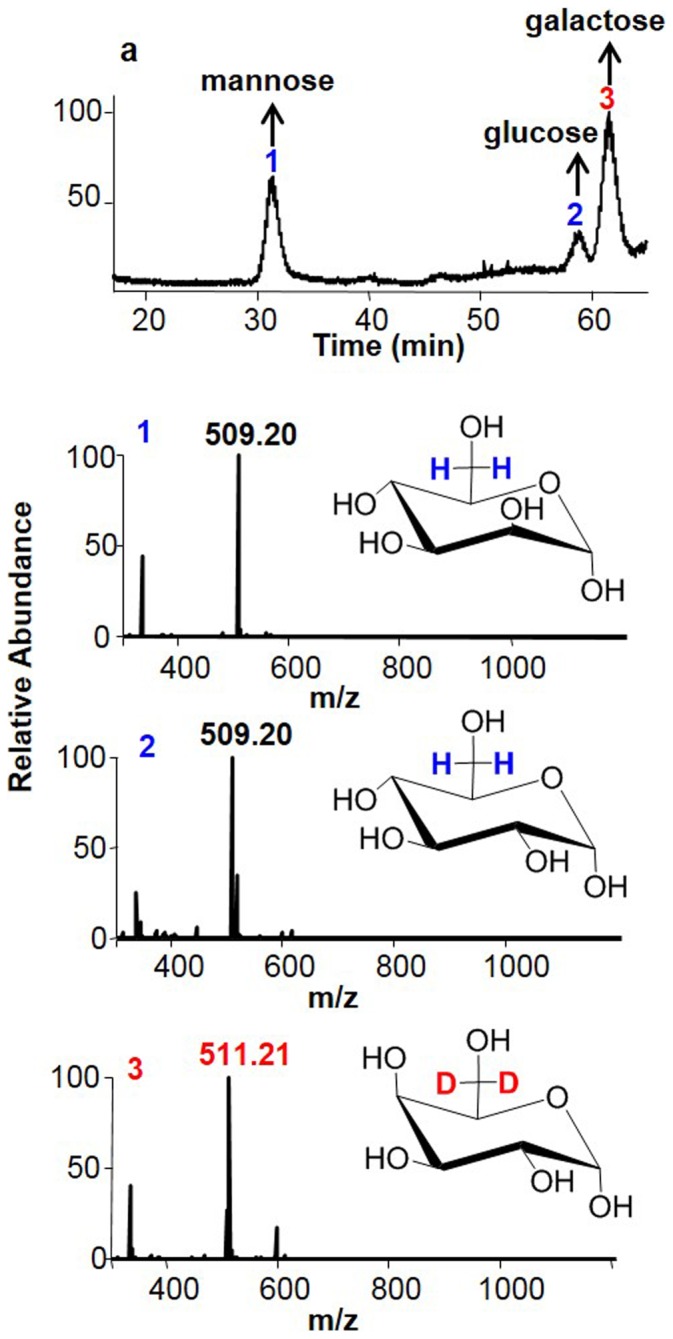
The molecular identity of uronic acid in fractions 6-8 (F6, F7, and F8). Five mg of EDC was added to 50 µl oligosaccharides F6-F8 mixture. As the reaction proceeded, the pH of the reaction mixture was maintained at 4.7 for 2 hrs. After hydrogen ion uptake had ceased, an aqueous 2 M NaBD_3_CN solution was added slowly to the reaction mixture at room temperature. The mixture was then hydrolyzed into monosaccharide according to the published protocol [35]. The hydrolyzed products were labeled with PMP and analyzed by capillary HPLC coupled MS (Details is in Methods).

To determine the molecular identity of the uronic acid, we took an aliquot from F6, F7, and F8, respectively, and then combined them. We first reduced the uronic acid in the mixture by using 1-ethyl-3-(3-dimethylaminopropyl) carbodiimide (EDC) and NaBD_3_CN followed by performing the monosaccharide composition analysis after acid hydrolysis and PMP derivatization. The results were shown in Fig. 6. Three kinds of PMP-derived monosaccharides were detected by both LC and MS: 1, mannose; 2, glucose, and 3, galactose by comparing to the elution positions and m/z of PMP-derived monosaccharide standards. The peak 3 of galactose was derived exclusively from galacturonic acid because of the unnatural molecular weight of 511.21, which could only be resulted from NaB**D**_3_CN reduction of galacturonic acid to form the 2x **D** containing galactose (Fig. 6).

Based on the relative abundances of the three monosaccharides, mannose, glucose, and galacturonic acid, we suspected the presence of (mannose)m-(glucose)n oligosaccharides in the combined F6, F7, and F8 fractions. Unfortunately, they could not be detected by MS directly for lacking negatively charged groups as in galacturonic acid- and sulfate-containing oligosaccharides as shown in Table 2.

We then used three different approaches including gel filtration (Fig. S3 in File S1), DEAE anion exchange HPLC (Fig. S4 in File S1), and cellulose acetate membrane electrophoresis (Fig. S5 in File S1) to purify polygalacturonic acid in P5. However, all three methods failed to show the existence of an unique polygalacturonic acid. These studies suggest that polygalaturonic acid might be part of the polysaccharides either through covalent bonding or through polysaccharide/polysaccharide interactions.

### Identifying covalently linked sulfate in P5

**Figure 7:**
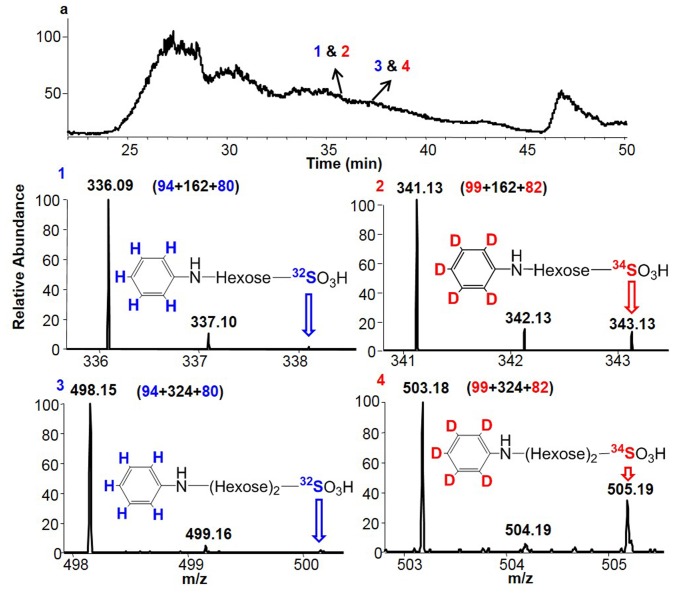
Identifying covalently linked sulfate in P5 and P5^34^S. The sulfated mono- and disaccharides were obtained by mild acid hydrolysis of P5 and P5^34^S that was carried out with 0.05 M TFA at 100°C for 3 hrs. The hydrolysates were dried and tagged with aniline for P5 or D_5_-aniline for P5^34^S (10 µl, 110 µmol) and then reduced with 10 µl of 1 M NaCNBH_3_ (Sigma-Aldrich, USA) at 65°C for 2 hrs. An equal amount of aniline and D_5_-aniline labeled P5 and P5^34^S products were combined and simultaneously analyzed by capillary HPLC coupled MS (Details is in Methods).

The elemental analysis result in Table 1 showed that all P0 to P5 contained trace amount of sulfur, indicating the polysaccharides might contain either covalently or non-covalently linked sulfates. The data presented in Table 2 suggested the presence of covalently linked sulfated (hexose)_3_ and sulfated (hexose)_4_ based on the calculated molecular weight and their detectability after direct MS injection of the oligosaccharide-containing fractions. Since there is no data to show the presence of covalently linked sulfate in polysaccharides purified from fungi during the past, we felt obligated to provide additional proof. To this end, we cultured *C. Militaris* by adding 500mg/L Na_2_^34^SO_4_ to the growth medium as we had previously done for a human cell line [36]. We then purified the polysaccharides, named P5^34^S, from the culture. We performed the direct structural comparison of P5 and P5^34^S by tagging the P5 with aniline and the P5^34^S with D_5_-aniline after increasing the intensity of partial TFA hydrolysis used for oligosaccharide preparation (Fig. 4). Because different isotope tags have no effect on chromatographic retention times but can be discriminated by a mass detector, the differentially isotope-tagged mono-sulfated hexose and mono-sulfated (hexose)_2_ derived from P5 and P5^34^S could be compared and confirmed simultaneously by using capillary HPLC coupled MS analysis. The results were shown in Fig. 7. A presumed mono-sulfated hexose was eluted at 35 min with m/z 336.09 (1) of an aniline tag from P5 and m/z 341.13(2) of a D_5_-aniline tag from P5^34^S. The presumed mono-sulfated (hexose)_2_ was eluted at 36.5 min with m/z 498.15(3) of an aniline tag from P5 and m/z 503.18 (4) of a D_5_-aniline tag from P5^34^S. By employing ^34^S-sulfate metabolic labeling of the *C. Militaris* in culture, the ^34^S-sulfate isotopic peaks at m/z 343.13 and 505.19 from P5^34^S were greatly augmented compared to the isotopic peaks at the m/z 338.10 and m/z 500.15 from that of P5. These data provided the direct evidence that the compounds 1 & 2 and 3 & 4 were mono-sulfated hexose and mono-sulfated (hexose)_2_ from P5 and P5^34^S, respectively.

## Discussion

The data in Figs. 1, 2, &3 showed that the salinity-induced anti-angiogenesis activities of polysaccharides from cultured *C. Militaris* corresponded to the multitude structural changes of the polysaccharides detected by ^13^C-NMR analysis. Furthermore, the newly established techniques of stable ^34^S-sulfate isotope labeling, aniline- and D_5_-aniline tagging, and stable isotope facilitated uronic acid-reduction coupled with LC-MS analysis revealed for the first time the existence of covalently linked sulfate and the presence of polygalacuronic acids in the polysaccharides purified from the salt added culture (Fig. 3, 4, 5, 6, & 7 and Table 2). These data clearly demonstrated that culturing *C. Militaris* with added salts changed the biosynthesis process and produce structurally and functionally very different polysaccharides, which indicated that salinity might serve as an epigenetic control factor that regulates the structures and functions of polysaccharides in the cultured fungus.

A conflict between the NMR (Fig. 3) and MS data (Table 2) was noticed in that the ^13^C-NMR spectrum of P5 should exhibit CH3 signals corresponding to methyl and acetyl groups at 22–24 ppm according to the MS data (Table. 2), but the ^13^C-NMR analysis did not show the presence of such signal (Fig. 3). There were several possibilities to explain the discrepancy: 1. the relative low abundance of polygalacturonic acid in the total polysaccharides based on the monosaccharide compositional analysis (Fig. S2 in File S1, Fig. 5 and Fig. 6); 2. The relative low abundance of methyl- and acetyl-modified polygalacturonic acid residues compared to that of unmodified polygalacturonic acid residues as shown in Fig. S6 (Fig. S6 in File S1); 3. It has been reported that even though the same amount of oversulfated chondroitin sulfate (OSCS) and oversulfated dermatan sulfate (OSDS) possesses the same numbers of acetyl groups, but the CH3 proton signal intensities of acetyl groups in OSDS are only half of that in OSCS [37]; 4. NMR could detect 0.1% OSCS in heparin based on the CH3 proton signal of acetyl groups in OSCS but could not detect 30% oversulfated heparan sulfate in heparin reported by two independent studies [38], [39]; 5. NMR failed to detect up to 50% polysaccharides in chitosan and PEGylated copolymers[40]. These results suggest NMR analysis of a polysaccharide mixture might not have the same authority as of small molecules in providing quantitative structural resolution.

Due to non-template driven biosynthesis of polysaccharides that results in great structural heterogeneities, the polysaccharide structures are difficult to study. The unambiguous proof of polysaccharide biological functions has usually been taken a conventional molecular biology approach by knocking out a gene either responsible for polysaccharide biosynthesis or responsible for polysaccharide sulfations [17], [41] in the post genomic era. Our polysaccharide structure-based approach presented in this manuscript would be a good complement to the gene-based studies [17], [41].Since the genome sequence of *C. Militaris* is available [42], we tried to find the salinity-induced genes responsible for the polysaccharide sulfation based on sequence homology search using several known mammalian genes without success. We suspected that the 1.547 billion year split of fungus from animals [43] might explain our failed bioinformatic approach. We are currently setting up a sulfotransferase assay to purify and clone the sulfotransferase(s) in the salt-induced *C. Militaris*. We believe that the expressions, regulations, epigenetic controls, and functions of genes responsible for polysaccharide biosynthesis and functions in *C. Militaris* should be explainable at molecular level in near future.

All living organisms and cells adjust to the changes in their environment by integrating environmental inputs to mediate homeostatic responses by making functional molecules through expressing different genes [44]. In this sense, wild Cordyceps is unique in that it not only grows inside the caterpillar body but also grows at low temperature and high attitude in Tibetan Plateau and the Himalayas. Therefore, it might be reasonable to compare the polysaccharide structures and functions with those of wild stocks by cultivating *C. Militaris* with 50% of the normal atmospheric oxygen and just above freezing temperature in addition to the added salts. Such studies might be able to obtain new information about the biosynthetic control of the polysaccharides and other pharmaceutical active ingredients in cultured *C. Militaris*. Eventually, it might be possible to make *C. Militaris*, *C. Sinensis, or* other medicinal fungal cultures to reach or to exceed the pharmaceutical potency of their wild stocks.

Unlike O-linked and N-linked glycans that primarily assist protein functions in eukaryote cells [45], the polysaccharides purified from C. *Militaris* had their own biological functions as shown in Fig. 1 & 2 and Fig. S1 (Fig. S1 in File S1). Up to date, over 20 different kinds of polysaccharide-based drugs have been approved and used world widely with an annual sale over $10 billion dollars. A handful of polysaccharide-based drugs have preceded through phases I, II, and III clinical trials and are used extensively and successfully in Asia to treat various cancers and other diseases [46]. Among them, 13 different kinds of polysaccharide-based drugs purified from 9 kinds of fungi and 4 kinds of plants have been approved by Chinese FDA and used clinically as anti-cardiovascular disease, anti-cancer, anti-aging, or immune system-regulating drugs. Moreover, an anti-thrombotic drug derived from brown algae polysaccharides have been approved and used both intravenously and orally in China for over 27 years [47]. Further, the life-saving drug heparin, a polysaccharide purified from animal tissues, remains as an essential and no-replaceable drug in modern medicine after 78 years of clinical use despite its inherent heterogeneity in both molecular structures and biological functions compared to small molecule and protein drugs [17]. Thus, the clinical applications of polysaccharide-based drugs are better known than the basic understandings of the chemical, biochemical, and biological properties as well as the biosynthetic pathways of such molecules.

Hundreds of biological functions of medicinal mushrooms and fungi have been reported including immunomodulating, antioxidant, antidiabetic, antihypercholesterolemia, antitumor, antiparasitic, antifungal, antibacterial, antiviral and hepatoprotective effects [46]. Most of reported biological functions are associated with polysaccharides. Compared to small molecule- and protein-based drugs, the advantages of polysaccharide-based drugs are their broad spectrum of therapeutic properties, relatively low toxicity, and less drug-resistant associated problems. The disadvantages of polysaccharide-based drugs are the inherited heterogeneity of their structures and functions and lack of tools to do proper structure analyses [28]. Thus, developing more biological assays and novel structural characterization methods such as shown in Fig. 1–7 might be critical in understanding the structural and functional information encoded in the polysaccharides. Such knowledge might be essential for polysaccharide-based drugs derived from the largely untapped and abundant resources, such as medicinal mushrooms, fungi, herbs, and algae, to enter the main stream of modern medicine not only in Asia but also in the rest of the world.

The structural and functional information of polysaccharides can be dynamically changed by assimilating environmental inputs, such as by adding salts into *C. Militaris* culture media as shown in Fig. 1 & 2. These findings indicated that the structural and functional information of polysaccharides was not directly encoded in genes [19], [48]. Co-incidentally, many studies suggest that the aging-related cardiovascular-, cancer-, diabetes-, and other complex human diseases might not be directly encoded in genes either, but caused mainly by aging-associated defects that throw the system out of balance in addition to pathogens and other environmental factors. Thus, when disease arises, genetic and genomic studies might not provide all the answers. In contrast, the dynamically complex information in polysaccharide structures might provide a clue. Therefore, solid structural studies are essential in fully understanding the biological functions of polysaccharides.

Polysaccharides are as essential for life as DNAs, RNAs, proteins, lipids, glycans, and metabolites of the biological system [49]. The dynamic structural and functional information of polysaccharides may be required to collaborate in order to keep the cells or whole living system in balance [50]. Perhaps it might be essential to understand the structures and functions of polysaccharides from all sources including Cordyceps before a complete understanding of the system biology and epigenetics of human beings become possible. If correct, understanding the molecular and functional diversity of the polysaccharides might be the key to unlocking our understanding life before the aging-related cardiovascular-, cancer-, diabetes-, and other complex human diseases can be prevented and cured in future.

## Materials and Methods

### Materials

*Cordyceps Militaries*, strain 11Y-6, was a generous gift from fungal specialist, Prof. X.L. Jiang (Ocean University of China, Qingdao, China). F12, McCoy's 5A and 1640 medium were obtained from Sigma-Aldrich (USA). Fetal bovine serum was purchased from GIBICO (USA). Growth factor-reduced BD Matrigel was purchased from Beckton Dickinson Labware (USA). Other reagents were obtained from Sigma-Aldrich (USA).

### Liquid culture of *C. Militaris*, polysaccharides extraction, and monosaccharide composition analysis

*C. Militaris* was maintained on potato dextrose agar (PDA) slants and inoculated at the center of a Petri dish. The mycelium was fermentation in 200 ml medium containing 6 g/L potato dextrose (PD) and 20 g/L glucose with 0.05 mg/L–500 mg/L Na_2_SO_4,_ 0.04 mg/L–410 mg/L NaCl, or 50 mg/L Na_2_^34^SO_4_ at 27°C for 10 days (130 r/min). The mycelia and culture medium were rapidly concentrated by rotary evaporation to 100 mL and extracted with 400 ml 95% ethanol at 80°C 3 times to remove lipids. The de-lipided power was extracted with water 3 hrs at room temperature (22°C) 3 times. The water soluble extracts were combined and then dialyzed against water in a bag with a molecular weight cut off 3000 Da membrane. The water soluble polysaccharides were precipitated by adding 4 volumes of 95% ethanol at 4°C overnight.

The polysaccharides P0-P6 were analyzed by elemental analysis, which was done by using a CHNS/O Analyzer. For monosaccharide composition analysis, polysaccharides (0.5 mg) were hydrolyzed with 2 M trifluoroacetic acid (TFA) at 110°C for 6 hrs. The hydrolyzed products were labeled by using 1-phenyl-3-methyl-5-pyrazolone (PMP) and analyzed by HPLC as described by Honda et al [35].

### Preparation and characterization of oligosaccharides

Partial hydrolysis of the P5 was carried out by using 0.05 M TFA at 100°C for 1.5 hrs. The hydrolysates were subjected to gel filtration chromatography using a Superdex 30 HPLC column. A 0.1 M NH_4_HCO_3_ solution at a flow rate of 0.1 mL/min was used for oligosaccharide elution. Each oligosaccharide fraction (F1 to F11) was monitored by an online refractive index detector and collected accordingly. An aliquot from each oligosaccharide fraction in 50% acetonitrile were directly infused into MS and detected by negative-ion ESI.

### Preparing sulfated mono- and disaccharides by mild acid hydrolysis

The sulfated mono- and disaccharides were obtained by mild acid hydrolysis that was carried out with 0.05 M TFA at 100°C for 3 hrs. The hydrolysates were dried and tagged with aniline or D_5_-aniline (10 µl, 110 µmol) and then reduced with 10 µl of 1 M NaCNBH_3_ (Sigma-Aldrich, USA) at 65°C for 2 hrs. An equal amount of aniline and D_5_-aniline labeled products were combined and simultaneously analyzed by capillary HPLC coupled MS.

### Reducing galacturonic acid to galactose in collected oligosaccharides

Five mg of EDC was added to 50 µl oligosaccharides F6-F8 mixture. As the reaction proceeded, the pH of the reaction mixture was maintained at 4.7 for 2 hrs. After hydrogen ion uptake had ceased, an aqueous 2 M NaBD_3_CN solution was added slowly to the reaction mixture at room temperature. The mixture was then hydrolyzed into monosaccharide according to the published protocol [35]. The hydrolyzed products were labeled with PMP and analyzed by capillary HPLC coupled MS as described below.

### Capillary HPLC coupled MS analysis

For PMP-labeled monosaccharide analysis, the capillary HPLC separations were performed using a solvent system composed of acetonitrile (eluent A) and 0.01 M CH_3_COONH_4_ in water pH 5.5 (eluent B). After injection of 0.5 µl samples, the elution profile was 17% A for 10 min, 19% A for 30 min, 21% A for 15 minutes, and 17% A for 25 minutes. The flow rate was 10 µl per minute, the absorbencies at 245 nm were monitored during each run and the negative ion spectra were collected by using an online Thermo LTQ-XL mass spectrometer.

The same elution condition for capillary HPLC separation of aniline-labeled products was used as described by Lu et al [51]. Briefly, an Agilent 1290 series capillary HPLC workstation was coupled with Thermo LTQ-XL mass spectrometer. Negative ion spectra were collected by scanning the m/z range 100–1000. Total ion current chromatograms were collected. The mass spectra were processed with Thermo Xcalibur software.

### ^3^C-NMR analysis

Each sample was dissolved in 500 µL D_2_O and freeze-dried twice to replace all exchangeable protons with deuterium. The ^13^C NMR spectrum was recorded on a Bruker DPX 400 spectrometer; acetone (the signals at 204.19 ppm and 30.17 ppm) served as an external reference.

### Cell culture

Human colorectal cancer cell lines (HCT116 and HT29), human lung cancer cell lines (H1299 and A549), and human umbilical vein endothelial cells (HUVECs) were obtained from Shanghai Cell Bank of Chinese Academy of Science and cultured in McCoy's 5A media supplemented with 5% FBS (HyClone), 1640 media supplemented with 5% FBS, or F12 media supplemented with 10% FBS (Gibco, USA), respectively.

### Cell proliferation assay

HCT116 (2000 cells/well), HT29 (2000 cells/well), A549 (1500 cells/well), H1299 (3000 cells/well) and HUVEC (2000 cells/well) cells were seeded in 96-well plates for 24 hrs. The monolayer was incubated with 100 µg/mL P0-P5 or P1C-P5C. Cells were cultured for 48 hrs, followed by adding 20 µl resazurin (2 mg/ml dissolved in water) to the media for 16 hrs. The fluorescent signal was monitored using 544 nm excitation wavelength and 595 nm emission wavelength by Spectramax M5 plate reader (Molecular Devices, USA).

### Capillary tube formation and transwell cell migration assays

Capillary tube formation assays were carried out as described by Qiu et al [52]. Briefly, HUVEC cells (3×10^4^ cells/well) were cultured on Matrigel in a 96-well plate for 24 hrs with 100 µg/mL of P0-P5, P1C-P5C or blank control. The enclosed capillary networks of tubes were photographed by a microscope (Olympus, CKX41, Japan) and the numbers of capillary tubes formed were counted at different time intervals.

The transwell cell migration assay was performed by using a 24-well chamber (Costar, Cambridge, MA, U.S.A.) as the outer chamber and polycarbonate filters (8 µm pores) as the inner chamber. HUVECs were starved overnight in serum-free F12 medium, harvested by trypsinization and 5×10^4^ cells were seeded into the inner chambers in F12 (1%FBS) media with 100 µg/mL P5 or blank control. The outer chambers contained the F12 media with 10% FBS. After incubation for 18 hrs at 37°C, the cells on the lower surface of the filter were fixed and stained with 0.1% crystal violet. Images of the migrated cells were taken using a microscope (Olympus, CKX41, Japan). The migrated cells were quantified at 595 nm after extraction with 10% acetic acid. Migrated cells in the outer chambers were also quantified independently by incubating the out chambers in F12 media with 10% FBS for 8 days followed by resazurin quantification.

### Protein extraction and western blot analysis

HUVEC cells were incubated with serial concentrations of P5 for 72 hrs. The cells were washed twice with ice-cold PBS and lysed in 50 µl 1× RIPA buffer containing 20 mM Tris-HCl,150 mM NaCl, 1 mM Na_2_EDTA, 1 mM EGTA, 1% Triton, 2.5 mM sodium pyrophosphate, 1 mM beta-glycerophosphate, 1 mM Na_3_VO_4_, 1 µg/ml leupeptin(pH 7.5). The insoluble pellet was discarded and protein concentration was determined by using BCA Protein Assay Kit (Beyotime, Beijing, China). Equal amount of proteins were loaded and fractionated by electrophoresis in a 15% polyacrylamide-SDS gel and transferred to nitrocellulose membrane. The membrane was incubated with indicated antibodies and then incubated with appropriate secondary antibodies. The resulting blots were visualized using enhanced chemiluminescence (ODYSSEY, LI-COR, USA). The numbers underneath of the blots represent band intensity measured by Image J software. β-Actin was served as an equal loading control. Antibodies for VEGF were obtained from SANTA CRUZ Biotechnology, USA (VEGF(A-20):SC-152). Antibodies for β-actin were purchased from Cell Signaling Technology (USA).

### Statistical Analysis

All data are represented as the mean±S.D. Comparison between groups was made by one-way analysis of variance (ANOVA) followed by a specific post hoc test to analyze the difference. P<0.05 was considered to indicate statistical significance.

## Supporting Information

File S1 **Supporting Figures. Figure S1. The effects of P1C-P5C on capillary tube formation of HUVECs on Metrigel (a & b) and growth inhibitory on HUVEC (c).** A 96-well plate coated with 55 µL Matrigel per well was allowed to solidify at 37°C for 30–60 min. HUVEC cells (3×10^4^ cells/well) were seeded and cultured in 200 µL F12 media containing 100 µg/mL of P1C-P5C or blank control for 4–12 hrs. After 4, 6, 8, 10, 12 hrs of incubation, the enclosed capillary networks of tubes were photographed at 8hrs by a microscope (a). The numbers of capillary tubes formed were counted (b). Growth inhibitory effect of P1C-P5C on HUVECs (c). The experiment was repeated twice with comparable results. **Figure S2. Monosaccharide compositions of P0-P5.** Each polysaccharide (0.5 mg) was hydrolyzed with 2 M trifluoroacetic acid (TFA) at 110°C for 6 h and the products were labeled with PMP and analysis by HPLC as described^36^. **Figure S3. The molecular weight-distribution of the P5 polysaccharide.** The data was collected by HPLC/GPC tandem DAWN HELEOS II. MALLS data was calculating by using ASTRA software. The horizontal axis represented the molar mass of the P5 polysaccharide, Y axis represented the size distribution of the polysaccharide. This result indicated that P5 could not be separated into unique polysaccharide by gel filtration chromatography. **Figure S4. DEAE-anion exchange HPLC analysis of P5 polysaccharide.** P5 was separated into two polysaccharide peaks. When each peak was collected and subjected to monosaccharide composition analysis after reduction with NaBD4, we found that most abundant monosaccharides in both peaks were mannose, galactose, and glucose. Galacturonic acid was enriched in peak II but it was also present in peak I. These results suggest that polygalaturonic acid might be part of the polysaccharides either through covalent bond or through polysaccharide/polysaccharide interactions. **Figure S5. Cellulose acetate membrane electrophoresis of P5, P5-E and glycosaminoglycans in pH 3.0 0.1 mol/L pyridine− 0.47 mol/L formic acid (7 mA, 20 min).** The membranes were stained with 0.5% Alcian blue in 2% acetic acid for 30 min and washed with 2% acetic acid for 30 min. P5-E were enzymatically extracted P5. CSA: chondroitin sulfate A; HEP: heparin; DS: dermatan sulfate; HS: heparan sulfate; CSC: chondroitin sulfate C. The migrating property during electrophoresis plus stainable by cation dye Alcian blue suggest the presence of acidic polysaccharides in P5. **Figure S6. MS analysis of F3.** The MS data indicated that methylated and acetylated uronic acid were present in relatively low abundances in F compared to the unmodified uronic acid.
(DOC)

## References

[1] Winkler D (2005) Yartsa Gunb'u—Cordyceps sinensis. Economy, ecology \& ethnomycologyof a fungus endemic to the Tibetan Plateau. Memorie dell Societa Italiana di Science Naturali e Del Museo Civico di Storai Naturale do Milano 33: 69–85.

[2] Das SK, Masuda M, Sakurai A, Sakakibara M (2010) Medicinal uses of the mushroom Cordyceps militaris: current state and prospects. Fitoterapia 81: 961–968. 20650308 10.1016/j.fitote.2010.07.010

[3] Shashidhar MG, Giridhar P, Udaya Sankar K, Manohar B (2013) Bioactive principles from Cordyceps sinensis: A potent food supplement – A review. Journal of Functional Foods 5: 1013–1030. 10.1016/j.jff.2013.04.018 PMC7104994 32288795

[4] Li SP, Yang FQ, Tsim KW (2006) Quality control of Cordyceps sinensis, a valued traditional Chinese medicine. J Pharm Biomed Anal 41: 1571–1584. 16504449 10.1016/j.jpba.2006.01.046

[5] Cleaver PD, Loomis-Powers M, Patel D (2004) Analysis of Quality and Techniques for Hybridization of Medicinal Fungus Cordyceps sinensis (Berk.)Sacc. (Ascomycetes). International Journal of Medicinal Mushrooms 6: 152.

[6] Hsu T, Shiao L, Hsieh C, Chang D (2002) A comparison of the chemical composition and bioactive ingredients of the Chinese medicinal mushroom DongChongXiaCao, its counterfeit and mimic, and fermented mycelium of Cordyceps sinensis. Food Chem 78: 463–469.

[7] Winkler D (2010) Cordyceps sinensis: A precious parasitic fungus infecting Tibet. Field Mycology 11: 60–67.

[8] Yu R, Song L, Zhao Y, Bin W, Wang L, et al (2004) Isolation and biological properties of polysaccharide CPS-1 from cultured Cordyceps militaris. Fitoterapia 75: 465–472. 15261384 10.1016/j.fitote.2004.04.003

[9] Wang M, Meng XY, Yang RL, Qin T, Wang XY, et al (2012) Cordyceps militaris polysaccharides can enhance the immunity and antioxidation activity in immunosuppressed mice. Carbohyd Polym 89: 461–466. 10.1016/j.carbpol.2012.03.029 24750744

[10] Yan F, Wang B, Zhang Y (2014) Polysaccharides from Cordyceps sinensis mycelium ameliorate exhaustive swimming exercise-induced oxidative stress. Pharm Biol 52: 157–161. 24047103 10.3109/13880209.2013.820197

[11] Ohta Y, Lee JB, Hayashi K, Fujita A, Park DK, et al (2007) In vivo anti-influenza virus activity of an immunomodulatory acidic polysaccharide isolated from Cordyceps militaris grown on germinated soybeans. J Agr Food Chem 55: 10194–10199. 17988090 10.1021/jf0721287

[12] Song C, Jeon YJ, Yang BK, Ra KS, Sung JM (1998) The anti-complementary activity of exo-polymers produced from submerged mycelial cultures of higher fungi with particular reference to Cordyceps militaris. Journal Of Microbiological and biotechnology 5: 536–539.

[13] Zhang G, Huang Y, Bian Y, Wong JH, Ng TB, et al (2006) Hypoglycemic activity of the fungi Cordyceps militaris, Cordyceps sinensis, Tricholoma mongolicum, and Omphalia lapidescens in streptozotocin-induced diabetic rats. Appl Microbiol Biot 72: 1152–1156. 10.1007/s00253-006-0411-9 16575562

[14] Lin Y, Chiang B (2008) Anti-tumor activity of the fermentation broth of Cordyceps militaris cultured in the medium of Radix astragali. Process Biochem 43: 244–250.

[15] Yoo HS, Shin JW, Cho JH, Son CG, Lee YW, et al (2004) Effects of Cordyceps militaris extract on angiogenesis and tumor growth. Acta Pharmacol Sin 25: 657–665. 15132834

[16] Jaeken J (2010) Congenital disorders of glycosylation. Ann N Y Acad Sci 1214: 190–198. 21175687 10.1111/j.1749-6632.2010.05840.x

[17] Zhang L (2010) Glycosaminoglycans in development, health and disease. United States: Elsevier Science & Technology Books.

[18] Bishop JR, Schuksz M, Esko JD (2007) Heparan sulphate proteoglycans fine-tune mammalian physiology. Nature 446: 1030–1037. 17460664 10.1038/nature05817

[19] Esko JD, Selleck SB (2002) Order out of chaos: assembly of ligand binding sites in heparan sulfate. Annu Rev Biochem 71: 435–471. 12045103 10.1146/annurev.biochem.71.110601.135458

[20] Zhang L, Schwartz JJ, Miller J, Liu J, Fritze LM, et al (1998) The retinoic acid and cAMP-dependent up-regulation of 3-O-sulfotransferase-1 leads to a dramatic augmentation of anticoagulantly active heparan sulfate biosynthesis in F9 embryonal carcinoma cells. J Biol Chem 273: 27998–28003. 9774414 10.1074/jbc.273.43.27998

[21] Miyamoto K, Asada K, Fukutomi T, Okochi E, Yagi Y, et al (2003) Methylation-associated silencing of heparan sulfate D-glucosaminyl 3-O-sulfotransferase-2 (3-OST-2) in human breast, colon, lung and pancreatic cancers. Oncogene 22: 274–280. 12527896 10.1038/sj.onc.1206146

[22] Tessema M, Yingling CM, Thomas CL, Klinge DM, Bernauer AM, et al (2012) SULF2 methylation is prognostic for lung cancer survival and increases sensitivity to topoisomerase-I inhibitors via induction of ISG15. Oncogene 31: 4107–4116. 22158045 10.1038/onc.2011.577 PMC3307938

[23] Freimoser FM, Screen S, Bagga S, Hu G, St LR (2003) Expressed sequence tag (EST) analysis of two subspecies of Metarhizium anisopliae reveals a plethora of secreted proteins with potential activity in insect hosts. Microbiology 149: 239–247. 12576597 10.1099/mic.0.25761-0

[24] Wang BJ, Won SJ, Yu ZR, Su CL (2005) Free radical scavenging and apoptotic effects of Cordyceps sinensis fractionated by supercritical carbon dioxide. Food Chem Toxicol 43: 543–552. 15721201 10.1016/j.fct.2004.12.008

[25] Xiong C, Xia Y, Zheng P, Shi S, Wang C (2010) Developmental stage-specific gene expression profiling for a medicinal fungus Cordyceps militaris. Mycology 1: 25–66.

[26] Li SP, Song ZH, Dong TTX, Ji ZN, Lo CK, et al (2004) Distinction of water-soluble constituents between natural and cultured Cordyceps by capillary electrophoresis. Phytomedicine 11: 684–690. 15636186 10.1016/j.phymed.2003.07.011

[27] Zeng Y, Han Z, Yu G, Zhang L (2014) Polysaccharides Purified from Wild Cordyceps Activates FGF2/FGFR1c Signaling. Journal of Ocean University of China (*accept*).

[28] Hu DJ, Cheong KL, Zhao J, Li SP (2013) Chromatography in characterization of polysaccharides from medicinal plants and fungi. J Sep Sci 36: 1–19. 23225747 10.1002/jssc.201200874

[29] Wang B, Wei M, Zhang L (2003) Studies on Structure and Properties of Water Soluble Polysaccharide from Fruiting Body of Cordyceps Militarvs(L.) Link. Chem Res Chinese U 19: 37–40.

[30] Yang W, Yin Y, Song L, Zhao Y, Yu R (2008) Determination of monosaccharide composition of polysaccharides in cultured Cordyceps militaris by high performance anion exchange chromatography with pulsed electrochemical detection. Chinese Traditional and Herbal Drugs 39: 531–535.

[31] Smiderle FR, Sassaki GL, Van Griensven LJ, Iacomini M (2013) Isolation and chemical characterization of a glucogalactomannan of the medicinal mushroom Cordyceps militaris. Carbohydr Polym 97: 74–80. 23769519 10.1016/j.carbpol.2013.04.049

[32] Iemitsu M, Maeda S, Jesmin S, Otsuki T, Miyauchi T (2006) Exercise training improves aging-induced downregulation of VEGF angiogenic signaling cascade in hearts. Am J Physiol Heart Circ Physiol 291: H1290–H1298. 16617130 10.1152/ajpheart.00820.2005

[33] Ferrara N (1999) Molecular and biological properties of vascular endothelial growth factor. J Mol Med (Berl) 77: 527–543. 10494799 10.1007/s001099900019

[34] Lu M, Cheng J, Lin C, Chang C (2010) Purification, structural elucidation, and anti-inflammatory effect of a water-soluble 1,6-branched 1,3-α-d-galactan from cultured mycelia of Poria cocos. Food Chem 118: 349–356.

[35] Honda S, Akao E, Suzuki S, Okuda M, Kakehi K, et al (1989) High-performance liquid chromatography of reducing carbohydrates as strongly ultraviolet-absorbing and electrochemically sensitive 1-phenyl-3-methyl-5-pyrazolone derivatives. Anal Biochem 180: 351–357. 2817366 10.1016/0003-2697(89)90444-2

[36] Dündar M, Müller T, Zhang Q, Pan J, Steinmann B, et al (2009) Loss of Dermatan-4-Sulfotransferase 1 Function Results in Adducted Thumb-Clubfoot Syndrome. Anticancer Res 85: 873–882. 10.1016/j.ajhg.2009.11.010 PMC2790573 20004762

[37] Pan J, Qian Y, Zhou X, Lu H, Ramacciotti E, et al (2010) Chemically oversulfated glycosaminoglycans are potent modulators of contact system activation and different cell signaling pathways. J Biol Chem 285: 22966–22975. 20418371 10.1074/jbc.M109.063735 PMC2906290

[38] Keire DA, Mans DJ, Ye H, Kolinski RE, Buhse LF (2010) Assay of possible economically motivated additives or native impurities levels in heparin by 1H NMR, SAX-HPLC, and anticoagulation time approaches. J Pharm Biomed Anal 52: 656–664. 20233649 10.1016/j.jpba.2010.02.019

[39] Pan J, Qian Y, Zhou X, Pazandak A, Frazier SB, et al (2010) Oversulfated chondroitin sulfate is not the sole contaminant in heparin. Nat Biotechnol 28: 203–207. 20212477 10.1038/nbt0310-203

[40] Novoa-Carballal R, Riguera R, Fernandez-Megia E (2013) Disclosing an NMR-invisible fraction in chitosan and PEGylated copolymers and its role on the determination of degrees of substitution. Mol Pharm 10: 3225–3231. 23822664 10.1021/mp400267m

[41] Samson SC, Ferrer T, Jou CJ, Sachse FB, Shankaran SS, et al (2013) 3-OST-7 Regulates BMP-Dependent Cardiac Contraction. PLoS Biol 11: e1001727. 24311987 10.1371/journal.pbio.1001727 PMC3849020

[42] Zheng P, Xia Y, Xiao G, Xiong C, Hu X, et al (2011) Genome sequence of the insect pathogenic fungus Cordyceps militaris, a valued traditional Chinese medicine. Genome biology 12: R116. 22112802 10.1186/gb-2011-12-11-r116 PMC3334602

[43] James TY, Kauff F, Schoch CL, Matheny PB, Hofstetter V, et al (2006) Reconstructing the early evolution of Fungi using a six-gene phylogeny. Nature 443: 818–822. 17051209 10.1038/nature05110

[44] Gut P, Verdin E (2013) The nexus of chromatin regulation and intermediary metabolism. Nature 502: 489–498. 24153302 10.1038/nature12752

[45] Varki A (1993) Biological roles of oligosaccharides: all of the theories are correct. Glycobiology 3: 97–130. 8490246 10.1093/glycob/3.2.97 PMC7108619

[46] Wasser SP (2011) Current findings, future trends, and unsolved problems in studies of medicinal mushrooms. Appl Microbiol Biotechnol 89: 1323–1332. 21190105 10.1007/s00253-010-3067-4

[47] Zeng Y, Han Z, Yang M (2013) An Overview of Marine Polysaccharide-Derived Drugs in China. Chinese Journal of Marine Drugs 32: 67–75.

[48] Vives RR, Seffouh A, Lortat-Jacob H (2014) Post-Synthetic Regulation of HS Structure: The Yin and Yang of the Sulfs in Cancer. Front Oncol 3: 331. 24459635 10.3389/fonc.2013.00331 PMC3890690

[49] Marth JD (2008) A unified vision of the building blocks of life. Nat Cell Biol 10: 1015–1016. 18758488 10.1038/ncb0908-1015 PMC2892900

[50] Springer SA, Gagneux P (2013) Glycan evolution in response to collaboration, conflict, and constraint. J Biol Chem 288: 6904–6911. 23329843 10.1074/jbc.R112.424523 PMC3591600

[51] Lu H, McDowell LM, Studelska DR, Zhang L (2010) Glycosaminoglycans in Human and Bovine Serum: Detection of Twenty-Four Heparan Sulfate and Chondroitin Sulfate Motifs Including a Novel Sialic Acid-modified Chondroitin Sulfate Linkage Hexasaccharide. Glycobiol Insights 2010: 13–28. 20657722 PMC2909113

[52] Qiu P, Guan H, Dong P, Guo S, Zheng J, et al (2011) The inhibitory effects of 5-hydroxy-3,6,7,8,3',4'-hexamethoxyflavone on human colon cancer cells. Mol Nutr Food Res 55: 1523–1532. 21648071 10.1002/mnfr.201100070 PMC3449327

