# Supplementary material for: Salinity-Induced Anti-Angiogenesis Activities and Structural Changes of the Polysaccharides from Cultured Cordyceps Militaris
Source: PLoS One. 2014 Sep 9;9(9):e103880. doi: 10.1371/journal.pone.0103880 (PMC4159134; doi:10.1371/journal.pone.0103880)
Supplement: File S1 — Supporting Figures. Figure S1. The effects of P1C-P5C on capillary tube formation of HUVECs on Metrigel (a & b) and growth inhibitory on HUVEC (c). A 96-well plate coated with 55 µL Matrigel per well was allowed to solidify at 37°C for 30–60 min. HUVEC cells (3×10^4^ cells/well) were seeded and cultured in 200 µL F12 media containing 100 µg/mL of P1C-P5C or blank control for 4–12 hrs. After 4, 6, 8, 10, 12 hrs of incubation, the enclosed capillary networks of tubes were photographed at 8hrs by a microscope (a). The numbers of capillary tubes formed were counted (b). Growth inhibitory effect of P1C-P5C on HUVECs (c). The experiment was repeated twice with comparable results. Figure S2. Monosaccharide compositions of P0-P5. Each polysaccharide (0.5 mg) was hydrolyzed with 2 M trifluoroacetic acid (TFA) at 110°C for 6 h and the products were labeled with PMP and analysis by HPLC as described36. Figure S3. The molecular weight-distribution of the P5 polysaccharide. The data was collected by HPLC/GPC tandem DAWN HELEOS II. MALLS data was calculating by using ASTRA software. The horizontal axis represented the molar mass of the P5 polysaccharide, Y axis represented the size distribution of the polysaccharide. This result indicated that P5 could not be separated into unique polysaccharide by gel filtration chromatography. Figure S4. DEAE-anion exchange HPLC analysis of P5 polysaccharide. P5 was separated into two polysaccharide peaks. When each peak was collected and subjected to monosaccharide composition analysis after reduction with NaBD4, we found that most abundant monosaccharides in both peaks were mannose, galactose, and glucose. Galacturonic acid was enriched in peak II but it was also present in peak I. These results suggest that polygalaturonic acid might be part of the polysaccharides either through covalent bond or through polysaccharide/polysaccharide interactions. Figure S5. Cellulose acetate membrane electrophoresis of P5, P5-E and glycosaminoglycans in pH 3.0 [file pone.0103880.s001.doc]

**Supplementary Figure 1. The effects of P1C-P5C on capillary tube formation of HUVECs on Metrigel (a & b) and growth inhibitory on HUVEC (c).**


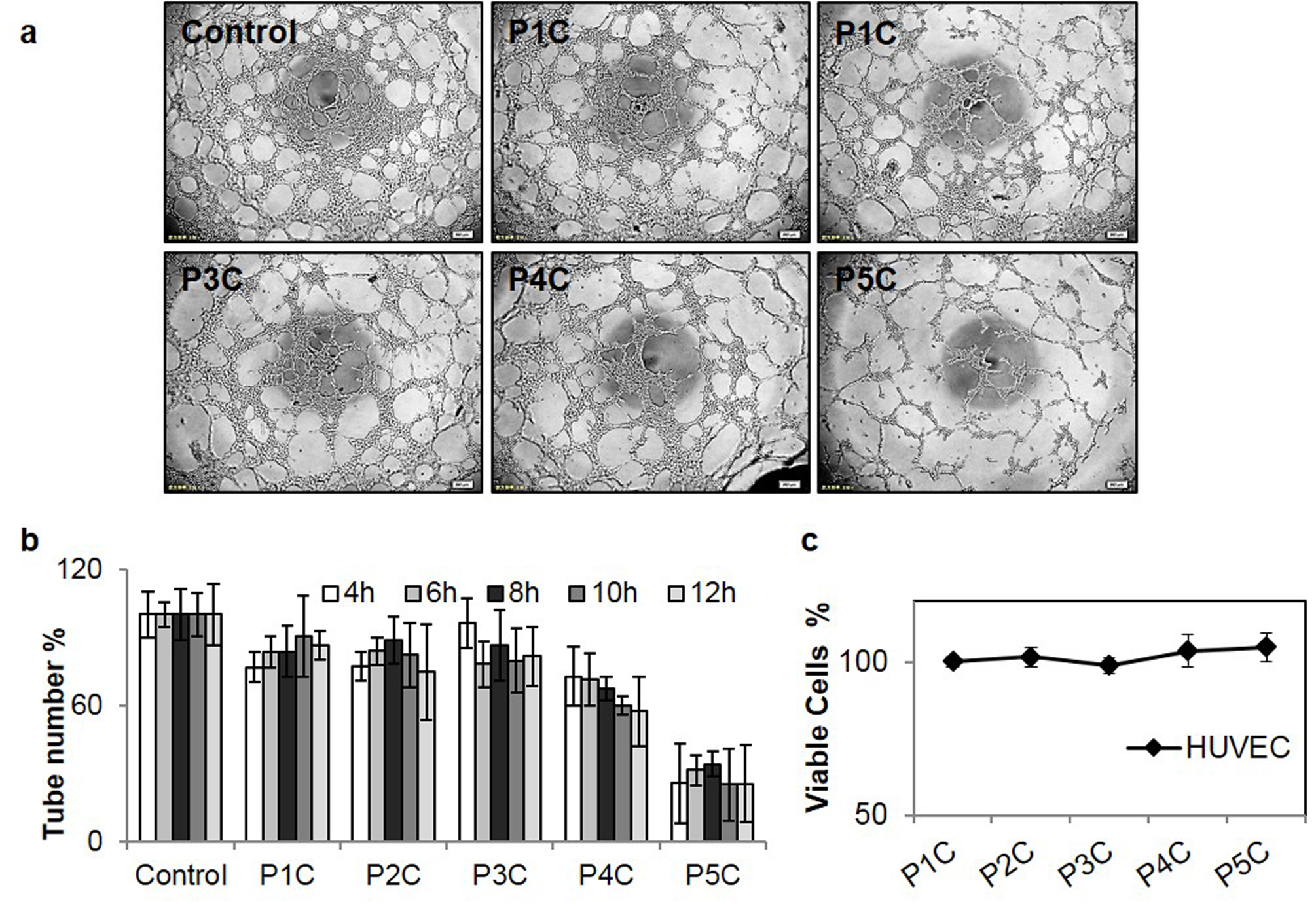


**Supplementary Figure 2. Monosaccharide compositions of P0-P5.**

**Supplementary Figure 3. The molecular weight-distribution of the P5 polysaccharide.**


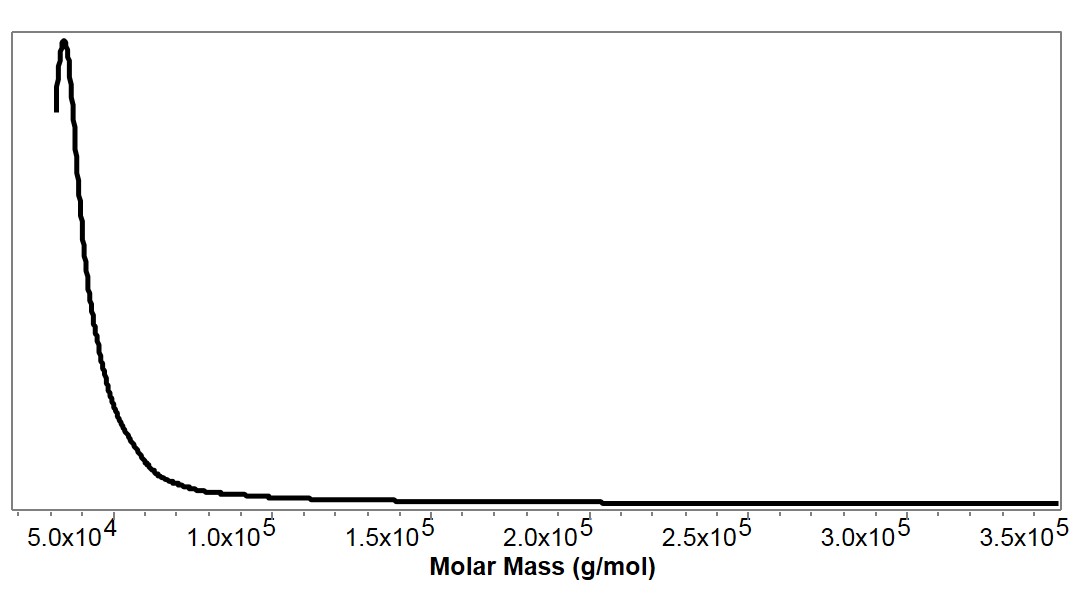


**Supplementary Figure 4. DEAE-anion exchange HPLC analysis of P5 polysaccharide.**

**
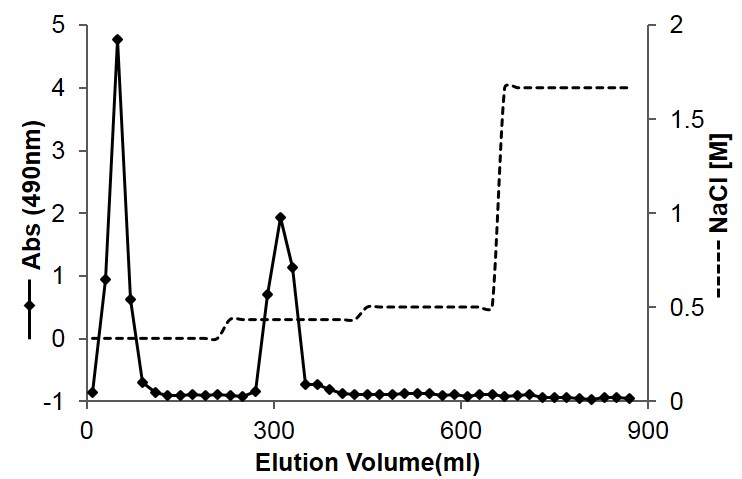
**

**Supplementary Figure 5.** **Cellulose acetate membrane electrophoresis of P5, P5-E and glycosaminoglycans in pH 3.0 0.1 mol/L pyridine- 0.47 mol/L formic acid (7 mA, 20 min).**

**
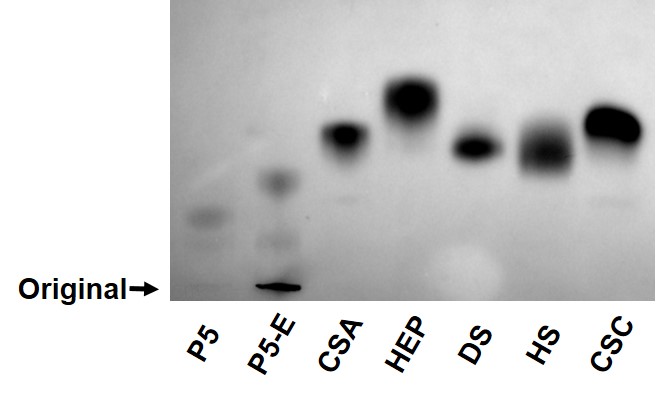
**

**Supplementary Figure 6.** **MS analysis of F3**.

**
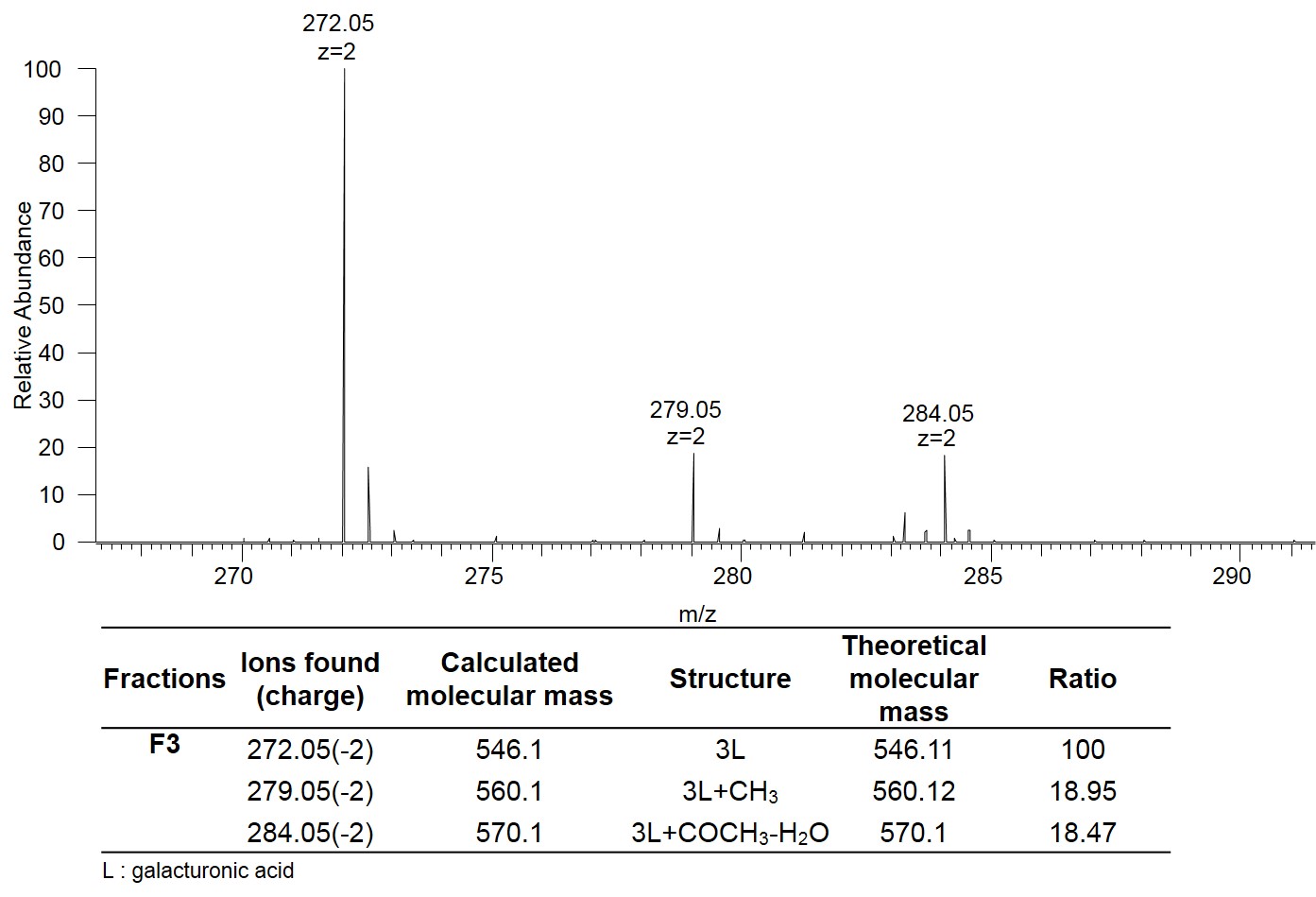
**
